# Supplementary material for: The Antimicrobial Peptide Cathelicidin Exerts Immunomodulatory Effects via Scavenger Receptors
Source: Int J Mol Sci. 2023 Jan 3;24(1):875. doi: 10.3390/ijms24010875 (PMC9821026; doi:10.3390/ijms24010875)
Supplement: Supplementary file 1 [file ijms-24-00875-s001.zip › ijms-1968452-supplementary.pdf]

International Journal of
*Molecular Sciences*

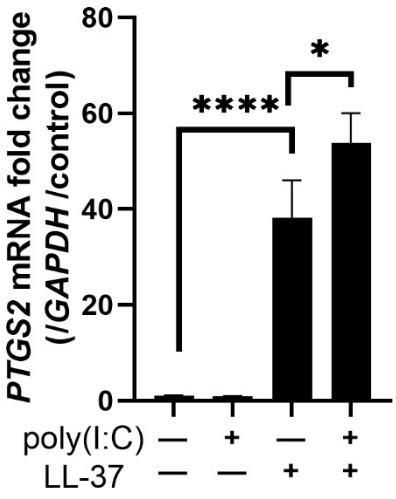


(**a**)

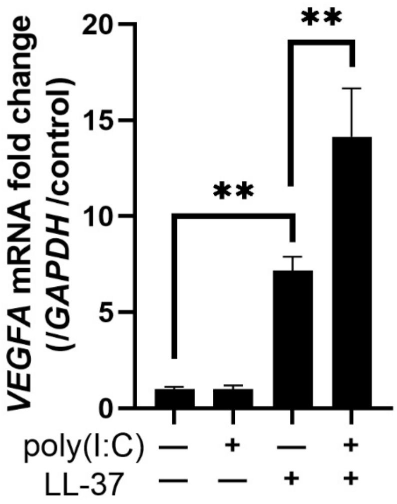


(**b**)

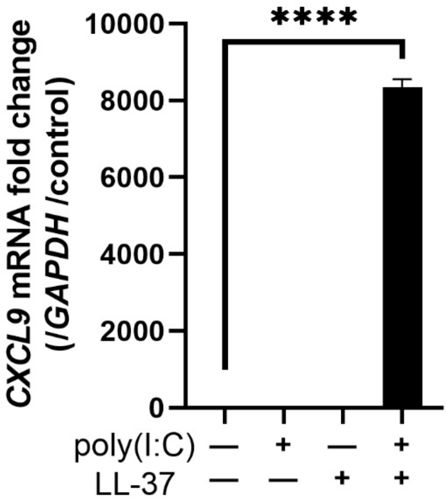


(**c**)

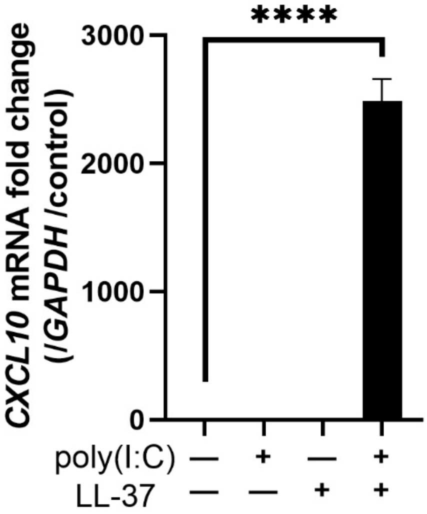


(**d**)

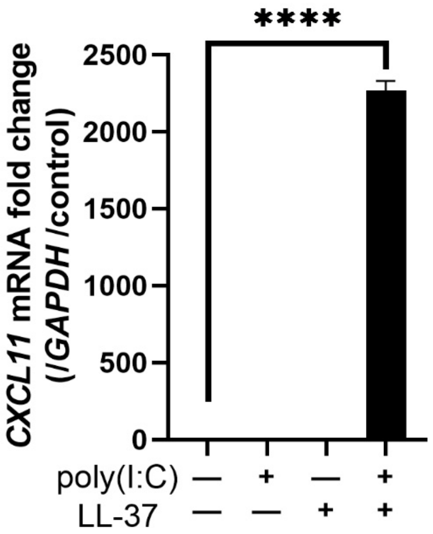


(**e**)

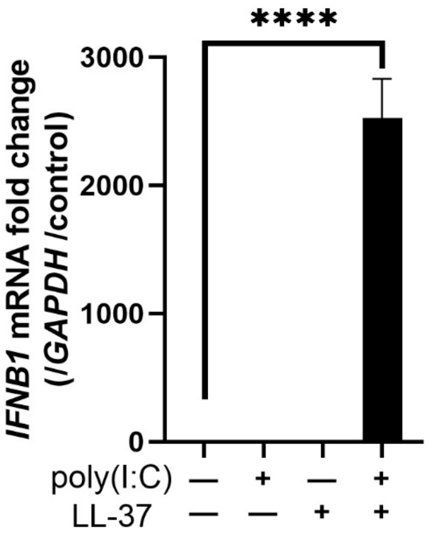


(**f**)

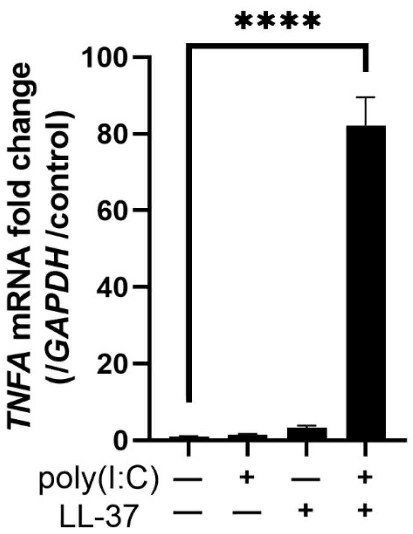


(**g**)

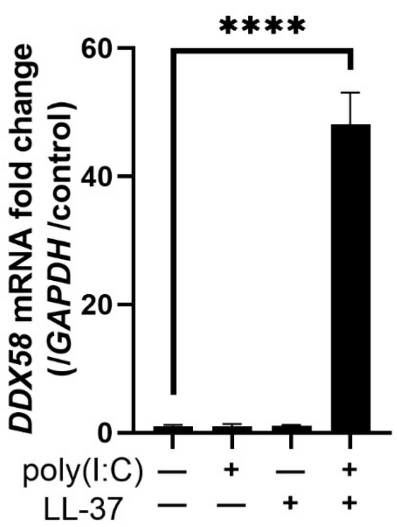


(**h**)

**Figure S1. Various mRNAs were induced by stimulation with LL-37 alone or in combination with poly(I:C) in keratinocytes.**

NHEKs were stimulated with LL-37 and poly(I:C) alone or in combination and incubated for 6 h. mRNA was extracted and relatively quantified by RT-PCR. (a) *PTGS2* and (b) *VEGFA* were mainly induced by LL-37 stimulation alone. (c) *CXCL9*, (d) *CXCL10*, (e) *CXCL11*, (f) *IFNB1*, (g) *TNFA,* and (h) *DDX58* were significantly induced by co-stimulation with LL-37 and poly(I:C). Data are means ± SEM of three biological replicates. * $p \leq 0.05$, **$p \leq 0.01$, ****$p \leq 0.0001$ by two-way ANOVA with Bonferroni's post hoc test.

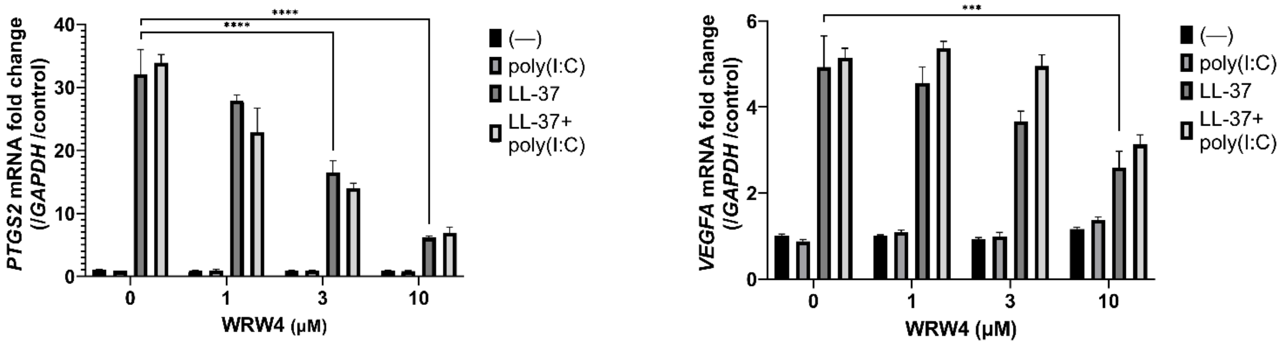


(**a**) (**b**)

**Figure S2. Induction of *PTGS2* and *VEGFA* by LL-37 was decreased depending on the dose of WRW4 in NHEKs.**

WRW4 (an FPR2 inhibitor) was applied to NHEKs at concentrations of 0–10 μM, and the cells were stimulated with LL-37 and poly(I:C) 1 h later. Cells were lysed and collected after 6 h of culture, and mRNA expressions of (a) *PTGS2* and (b) *VEGFA* were relatively quantified by RT-PCR. Data are means ± SEM of three biological replicates. ***p ≤ 0.001, ****p ≤ 0.0001 by two-way ANOVA with Bonferroni's post hoc test.

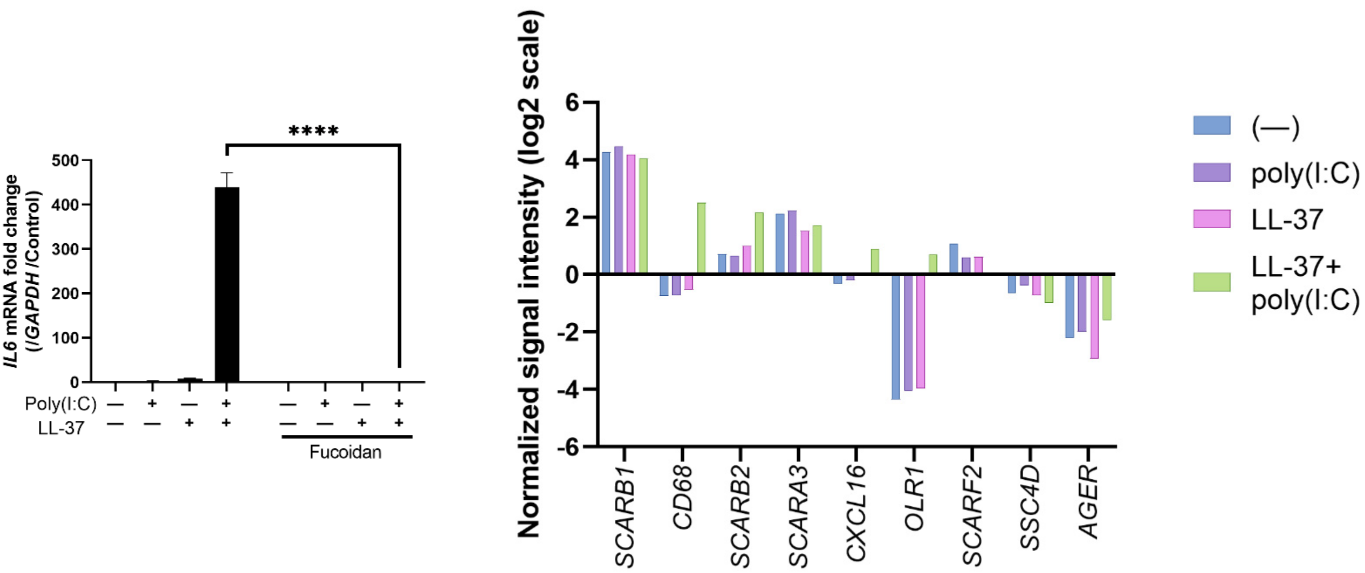


(**a**) (**b**)

**Figure S3. Signal intensities of various scavenger receptors in DNA microarrays.**

(a) NHEKs were stimulated with LL-37 alone or in combination with poly(I:C), with or without fucoidan, an inhibitor for multiple scavenger receptors, at a final concentration of 10 μg/ml for 6 h. mRNA was extracted and relatively quantified by RT-PCR. (b) NHEKs were stimulated with LL-37 and poly(I:C) alone or in combination and analyzed by DNA microarray. The signal intensities of scavenger receptors were examined, and the top 9 genes are shown. The expressions of *SCARB1* (SR-B1), *CD68* (SR-D1), *SCARB2* (SR-B2), *SCARA3* (SR-A3), *CXCL16* (SR-PSOX), *OLR1* (SR-E1), *SCARF2* (SR-F2), SSC4D, and AGER (SR-J1) are shown in order of signal intensity when stimulated with LL-37 and poly(I:C).

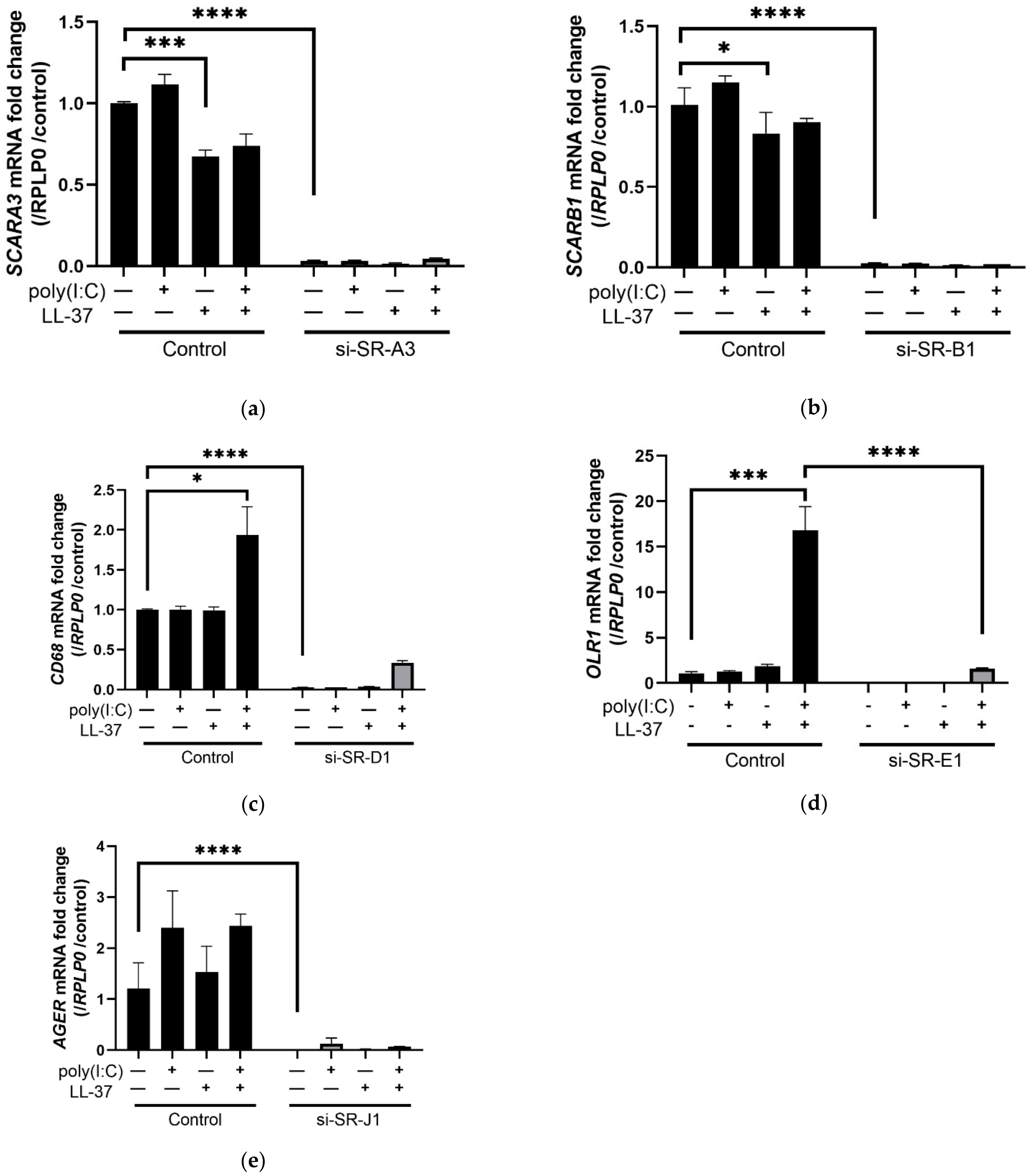


**Figure S4. Expressions of scavenger receptors were decreased by transfection with their siRNAs.**

NHEKs were transfected with siRNAs of various scavenger receptors, stimulated with LL-37 and poly(I:C), and RT-PCR analysis was carried out. Samples were the same as in Figure 3a-e. The mRNA expressions of (a) *SCARA3* (SR-A3), (b) *SCARB1* (SR-B1), (c) *CD68* (SR-D1), (d) *OLR1* (SR-E1), and (e) *AGER* (SR-J1) are shown. Data are means ± SEM of three biological replicates. NS, $p > 0.05$, * $p \leq 0.05$, **$p \leq 0.01$, ***$p \leq 0.001$, ****$p \leq 0.0001$ by two-way ANOVA with Bonferroni's post hoc test.

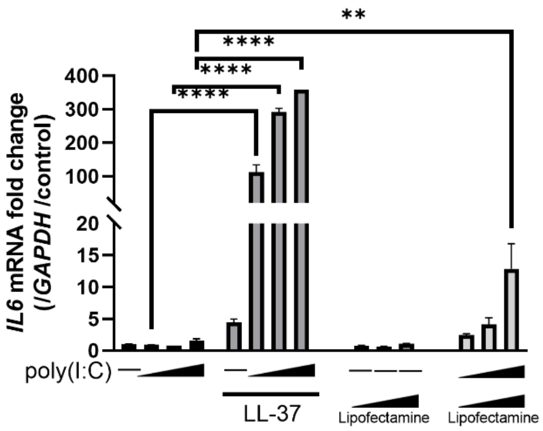


(**a**)

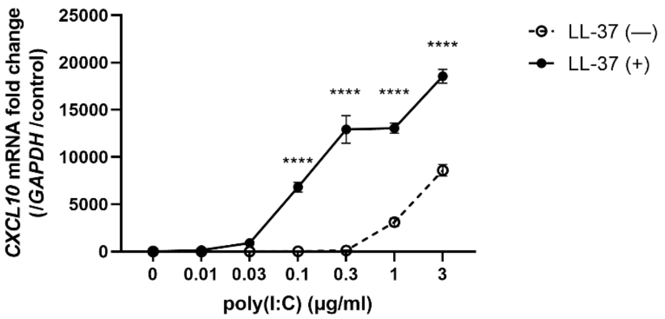


(**b**)

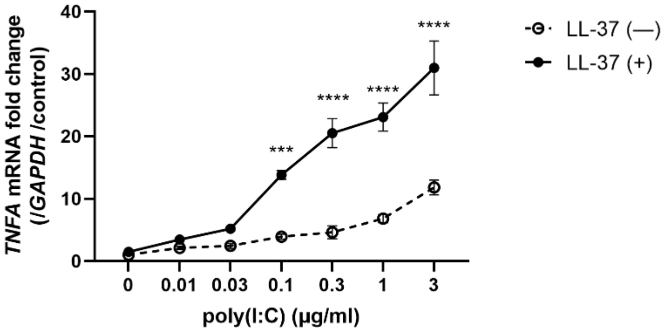


(**c**)

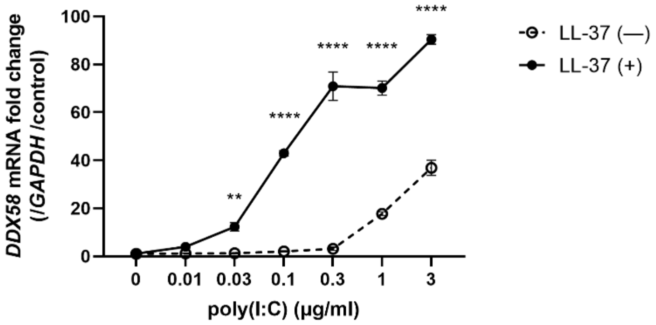


(**d**)

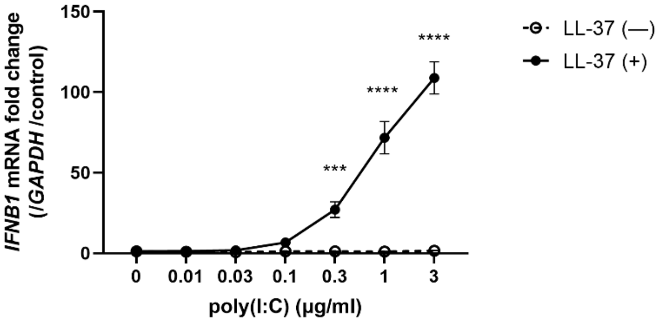


(**e**)

**Figure S5. Poly(I:C) induced cytokines in combination with a transfection reagent or at high concentration, but to a weaker extent than with LL-37.**

(a) NHEKs were stimulated with poly(I:C) and LL-37 or Lipofectamine 3000 reagent alone or in combination. Poly(I:C) was used at 0.1, 0.3, and 1 µg/ml, and Lipofectamine 3000 reagent was premixed and administered according to the amount of poly(I:C) used. The induction of *IL6* mRNA after 6 hours of stimulation was shown. Data are means ± SEM of two biological replicates. NHEKs were stimulated with poly(I:C) at different concentrations (0.01–3 µg/ml) alone or in combination with LL-37. RT-PCR was performed to quantify the mRNA expressions of (b) *CXCL10*, (c) *TNFA*, (d) *DDX58*, and (e) *IFNB1*. Data are means ± SEM of three biological replicates. **p ≤ 0.01, ***p ≤ 0.001, ****p ≤ 0.0001 by two-way ANOVA with Bonferroni's post hoc test.

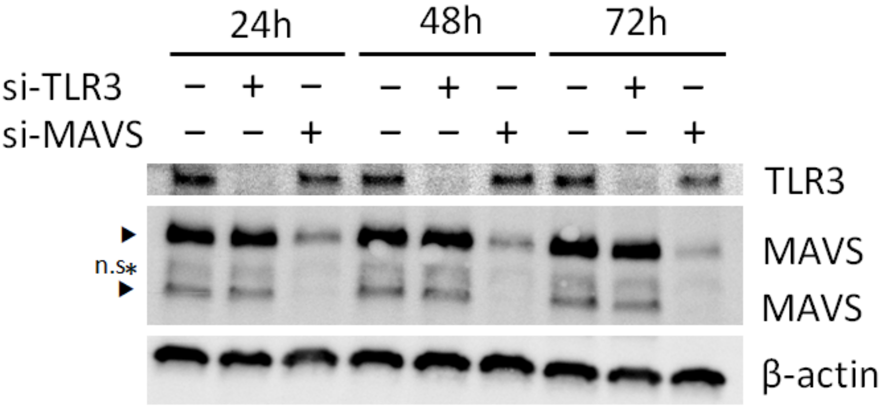


(**a**)

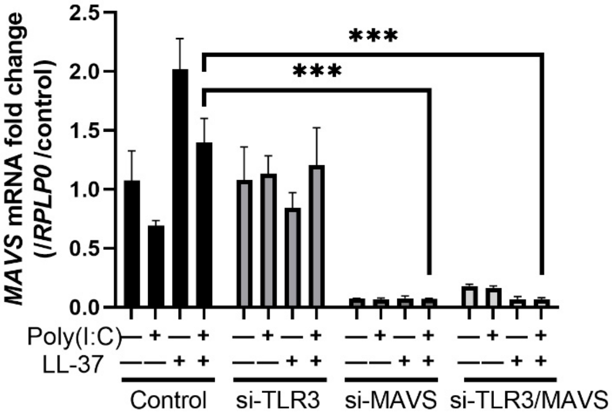


(**b**)

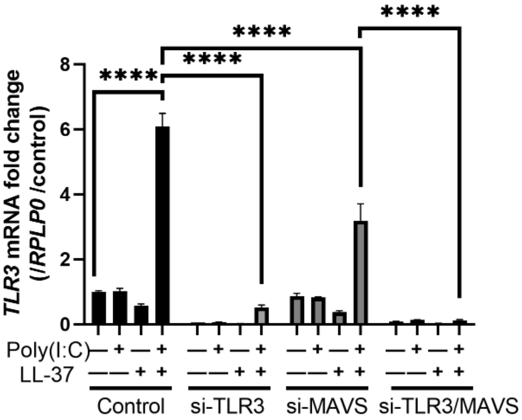


(**c**)

**Figure S6. siRNAs of TLR3 and MAVS were transfected into NHEKs.**

(a)NHEKs were transfected with control, TLR3 or MAVS siRNA for 24-72 hours, medium was exchanged, and samples were collected after 24 hours. Immunoblotting of TLR3 and MAVS were shown. β-actin was used as a loading control. NHEKs were transfected with TLR3 and MAVS siRNAs alone or in combination for 24 hours and stimulated with LL-37 and poly(I:C). Samples were the same as in Figures 4i-k. Expression of (b) *MAVS* and (c) *TLR3* mRNA was analyzed by RT-PCR. Data are means ± SEM of three biological replicates. NS, $p > 0.05$, * $p \leq 0.05$, ***$p \leq 0.001$, by two-way ANOVA with Bonferroni's post hoc test.

**Table S1. List of antibodies.**

| Antibodies | Origin | Source | Identifier | Dilution |
|---|---|---|---|---|
| CD68 (SR-D1) | Rabbit | Abcam | Cat#ab125212 | 1:500 |
| LL-37 | Mouse | Santa Cruz | Cat#sc-166770 | 1:200 |
| LOX1 (SR-E1) | Rabbit | Abcam | Cat#ab60178 | 1:100 |
| RAGE (SR-J1) | Rabbit | Abcam | Cat#ab3611 | 1:100 |
| SCARA3 (SR-A3) | Rabbit | Sigma | Cat#HPA047386 | 1:200 |
| SR-B1 | Rabbit | Novus Biologicals | Cat#NB400-104 | 1:200 |
| β-actin HRP conjugate | Rabbit | Cell signaling | Cat#5125 | 1:1000 |
| IRF-3 | Rabbit | Cell signaling | Cat#4302 | 1:1000 |
| MAVS | Rabbit | Cell signaling | Cat#3993 | 1:1000 |
| p38 | Rabbit | Cell signaling | Cat#9212 | 1:500 |
| Phospho-IRF-3 | Rabbit | Cell signaling | Cat#4947 | 1:1000 |
| Phospho-p38 | Rabbit | Cell signaling | Cat#9211 | 1:1000 |
| Phospho-TBK1 | Rabbit | Cell signaling | Cat#5483 | 1:1000 |
| TLR3 | Rabbit | Cell signaling | Cat#6961 | 1:1000 |
| TBK1 | Rabbit | Cell signaling | Cat#3504 | 1:1000 |
| Rabbit IgG, HRP-linked | Goat | Cell signaling | Cat#7074 | 1:1000 |
| Biotin, HRP-Linked | Goat | Cell signaling | Cat#7075 | 1:1000-3000 |
| IRDye® 800CW anti-Rabbit IgG | Donkey | LI-COR | Cat#926-32213 | 1:3000 |
| Duolink® In Situ PLA® Probe Anti-Mouse MINUS | Donkey | Sigma | Cat#DUO92004 | N/A |
| Duolink® In Situ PLA® Probe Anti-Rabbit PLUS | Donkey | Sigma | Cat#DUO92002 | N/A |

**Table S2. Assay ID of quantitative PCR primers and probes.**

| Gene | Reference sequence | Taqman® Gene Expression Assay ID |
|---|---|---|
| *CD68* | NM_001040059.1<br>NM_001251.2 | Hs02836816_g1 |
| *CXCL9* | NM_002416.2 | Hs00171065_m1 |
| *CXCL10* | NM_001565.3 | Hs00171042_m1 |
| *CXCL11* | NM_001302123.1<br>NM_005409.4 | Hs00171138_m1 |
| *DDX58* | NM_014314.3 | Hs01061436_m1 |
| *IFNB1* | NM_002176.3 | Hs01077958_s1 |
| *IL6* | NM_000600.4 | Hs00985639_m1 |

| | | |
|---|---|---|
| *IL36G* | NM_001278568.1<br>NM_019618.3 | Hs00219742_m1 |
| *MAVS* | NM_001206491.1<br>NM_020746.4 | Hs00920075_m1 |
| *OLR1* | NM_001172632.1<br>NM_001172633.1<br>NM_002543.3 | Hs01552593_m1 |
| *PTGS2* | NM_000963.3 | Hs00153133_m1 |
| *AGER* | NM_001136.4<br>Other 8 sequences | Hs00542584_g1 |
| *RPLP0* | NM_001002.3<br>NM_053275.3 | Hs99999902_m1 |
| *SCARA3* | NM_016240.2<br>NM_182826.1 | Hs00939871_m1 |
| *SCARB1* | NM_001082959.1<br>NM_005505.4 | Hs00969821_m1 |
| *TLR3* | NM_003265.2 | Hs01551078_m1 |
| *TNFA* | NM_000594.3 | Hs00174128_m1 |
| *VEGFA* | NM_001025366.2<br>Other 19 sequences | Hs00900055_m1 |

| Gene | Reference sequence | Primer sequence (5′-3′) | Probe sequence (5′-3′) |
|---|---|---|---|
| *GAPDH* | NM_002046 | GAAGGTGAAGGTCGGAGTC<br>GAAGATGGTGATGGGATTTC | TGGCAAATTCCATGGCAC-CGTCA |
